# 1427. The EpiRate: a multidimensional index for measuring effectiveness in infection prevention

**DOI:** 10.1093/ofid/ofad500.1264

**Published:** 2023-11-27

**Authors:** Braulio Couto, Ana Paula Ladeira, Walisson Ferreira Carvalho, Naísses Zóia Lima, Carlos E Starling

**Affiliations:** Biobyte Tecnologia em Epidemiologia, Belo Horizonte, Minas Gerais, Brazil; Biobyte Tecnologia em Epidemiologia, Belo Horizonte, Minas Gerais, Brazil; PUC MInas, Belo Horizonte, Minas Gerais, Brazil; PUC MInas, Belo Horizonte, Minas Gerais, Brazil; Sociedade Mineira de Infectologia - SMI, Belo Horizonte, Minas Gerais, Brazil

## Abstract

**Background:**

Measuring the effectiveness of infection prevention in a hospital setting is a crucial task, particularly from a global perspective and when considering various types of infections simultaneously (Fig. 1). In this paper, we introduce the EpiRate, a multidimensional index designed to assess the effectiveness of a hospital-wide ICP.

**Figure 1.**
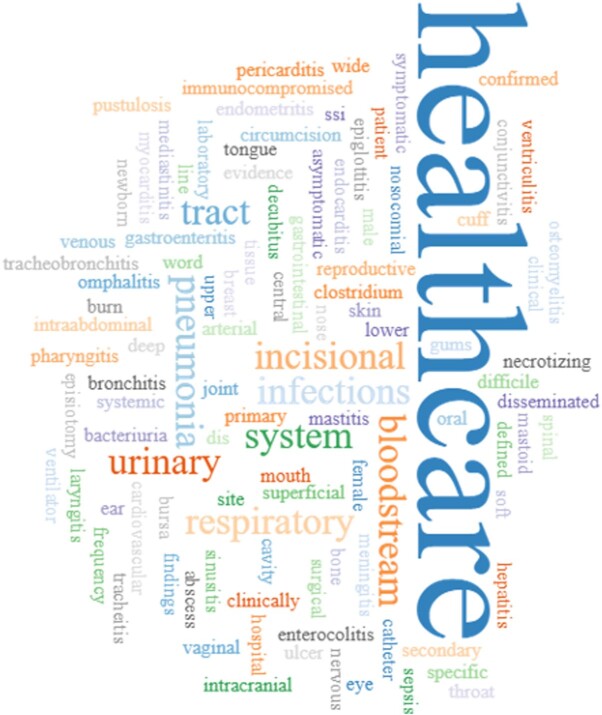
There are many types of hospital-acquired infections. How to measure the effectiveness in preventing hospital-acquired infection in a global perspective?

**Methods:**

EpiRate is calculated using a scoring system based on a comparison of observed values obtained from a list of hospital-acquired infection rates with the percentiles of their respective benchmarks. For each rate, the observed rate (Tx) is compared with the benchmark percentiles to assign a score. If Tx ≤ p10%, the score is set to 5.00; if Tx ≤ p25%, the score is 4.75; if Tx ≤ p50%, the score is 4.00; if Tx ≤ p75%, the score is 3.75; if Tx ≤ p90%, the score is 3.00; and if Tx > p90%, the score is 0.00 (Fig. 2). The total score is the sum of all points obtained considering the k rates involved in the analysis. The EpiRate is then calculated as the total score divided by the maximum score (= k*5), expressed as a percentage (%), indicating the percentage of effectiveness in preventing the infections measured by the chosen indicators. Furthermore, the prevention effectiveness of each infection can be assessed individually based on the indicators used in the EpiRate, with automatic interpretation (Fig. 3).

**Figure 2.**
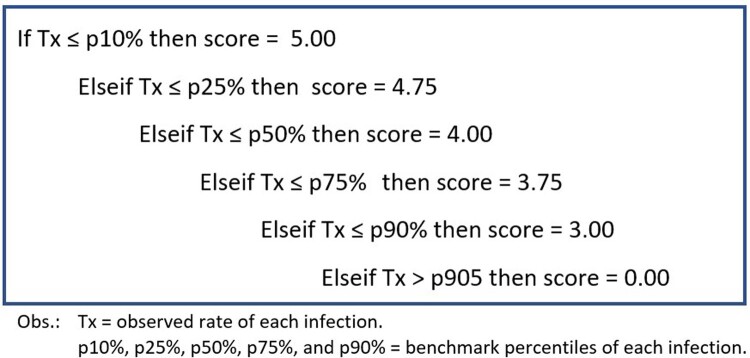
EpiRate Scoring Algorithm for Each Rate or Infection Considered

**Figure 3.**
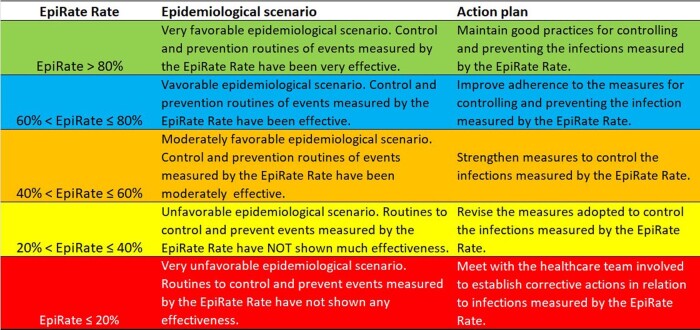
Epidemiological scenarios and action plans associated with the values of EpiRate rate.

**Results:**

Fig. 4 presents an example of the EpiRate for an ICU, utilizing 5 rates in the calculation (k=5). For each rate, observed values are compared with the benchmark percentiles, and the maximum score is 5 * 5 = 25 points. In this example, a total score of 19.5 points was obtained, resulting in an effectiveness rating of 78% for preventing infections in the ICU, from a global perspective. Fig. 5 illustrates the monthly EpiRate trends for two ICUs, from Jan/2021 to Dec/2022. Furthermore, Figure 6 showcases EpiRates specifically for Surgical Site Infections (SSIs), allowing an evaluation of the SSI prevention effectiveness in each service and enabling a summary of the hospital-wide SSI EpiRate.

**Figure 4.**
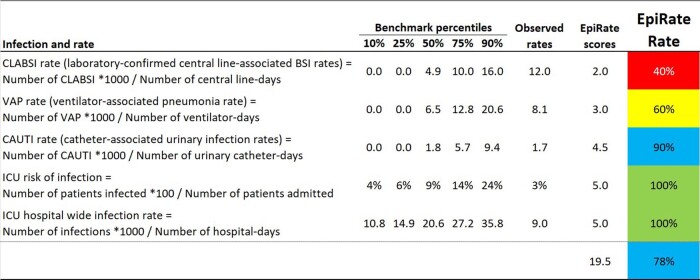
Example of a EpiRate Rate calculating in an Intensive Care Unit (ICU).

**Figure 5.**
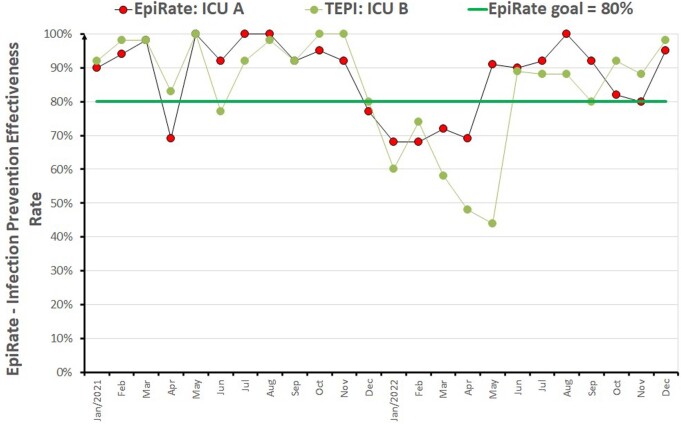
EpiRate for two ICUs (Jan/2021 to Dec/2022): monthly rates.

**Figure 6.**
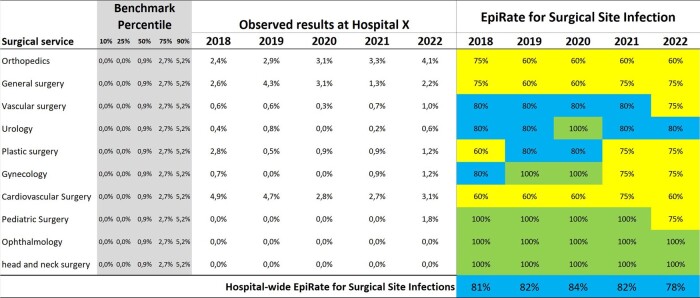
EpiRate for Surgical Site Infection.

**Conclusion:**

Unlike traditional infection rates that focus on the "bad side of the problem," EpiRate Rate provides a positive perspective by evaluating the effectiveness of infection control program. This multidimensional index has the potential to enhance infection prevention strategies.

**Disclosures:**

**All Authors**: No reported disclosures

